# Supplementary figures and images for: Co-crystallisation and humanisation of an anti-HER2 single-domain antibody as a theranostic tool
Source: PLoS One. 2023 Jul 17;18(7):e0288259. doi: 10.1371/journal.pone.0288259 (PMC10351726; doi:10.1371/journal.pone.0288259)

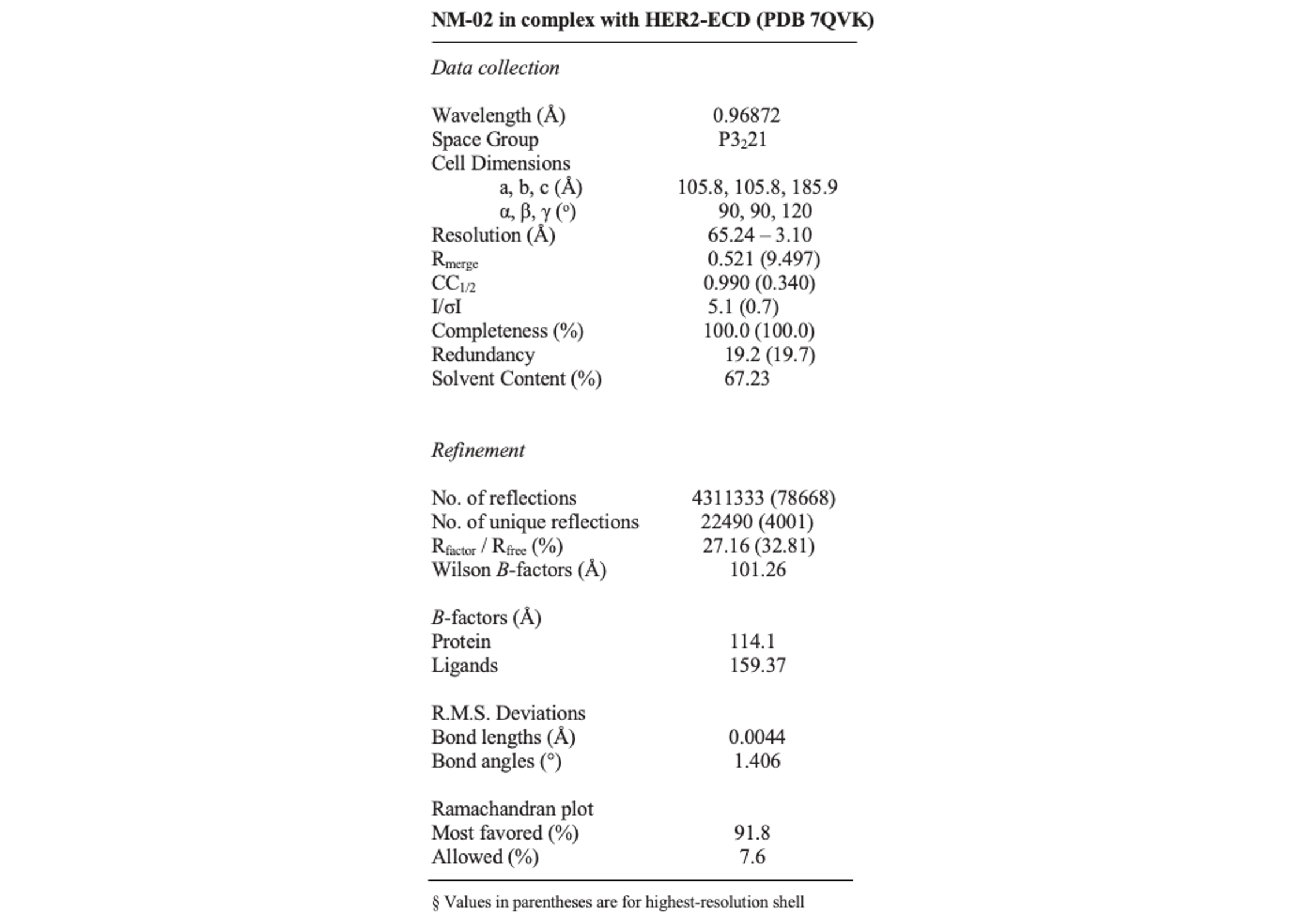

Supplement: S1 Table — (TIF) [file pone.0288259.s001.tif]

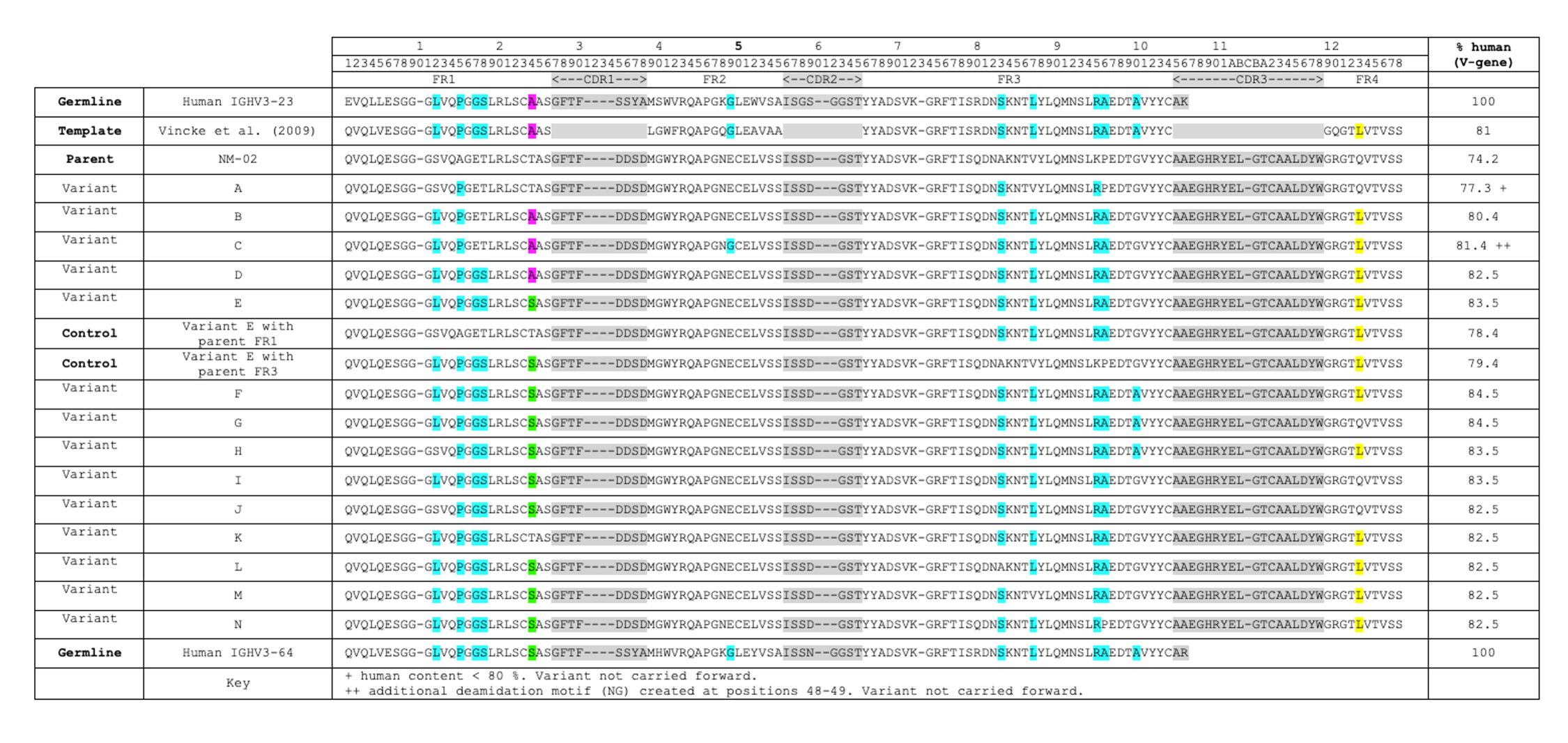

Supplement: S2 Table — The primary sequence for NM-02 was numbered according to IMGT [16] and inspected against a published template, human germline(s) and the experimentally-determined crystal structure (see Fig 3). Fourteen humanised variants (A to N) were designed for this study. Human germline residues incorporated into each NM-02 variant are coloured blue (common to both IGHV3-23 and IGHV3-64), pink (unique to IGHV3-23) or green (unique to IGHV3-64) respectively. A single non-germline residue (position 123; framework 4) was also examined (yellow). The corresponding human identity (%) of each NM-02 humanised variant is also shown. (TIF) [file pone.0288259.s002.tif]

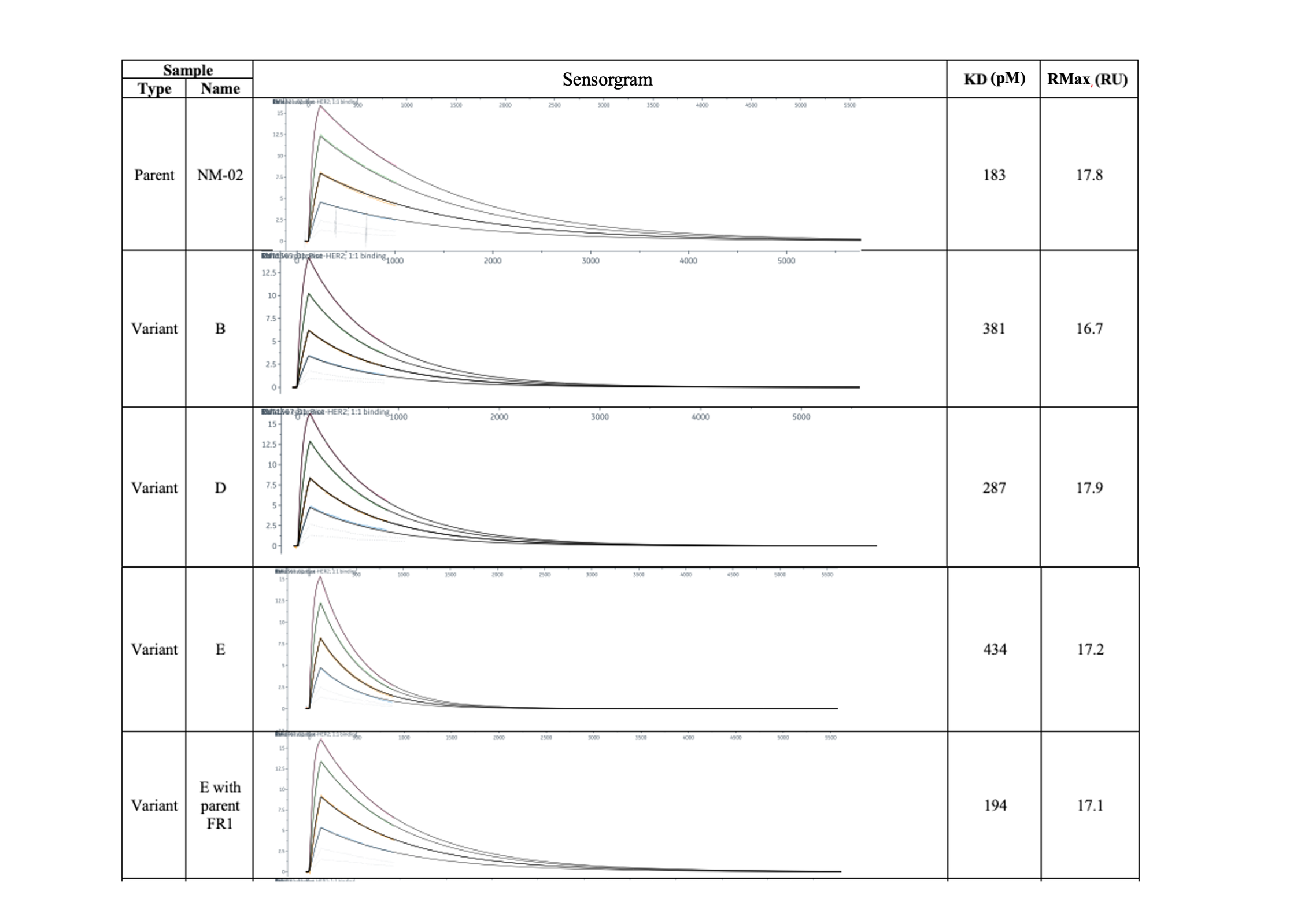

Supplement: S3 Table — (TIF) [file pone.0288259.s003.tif]

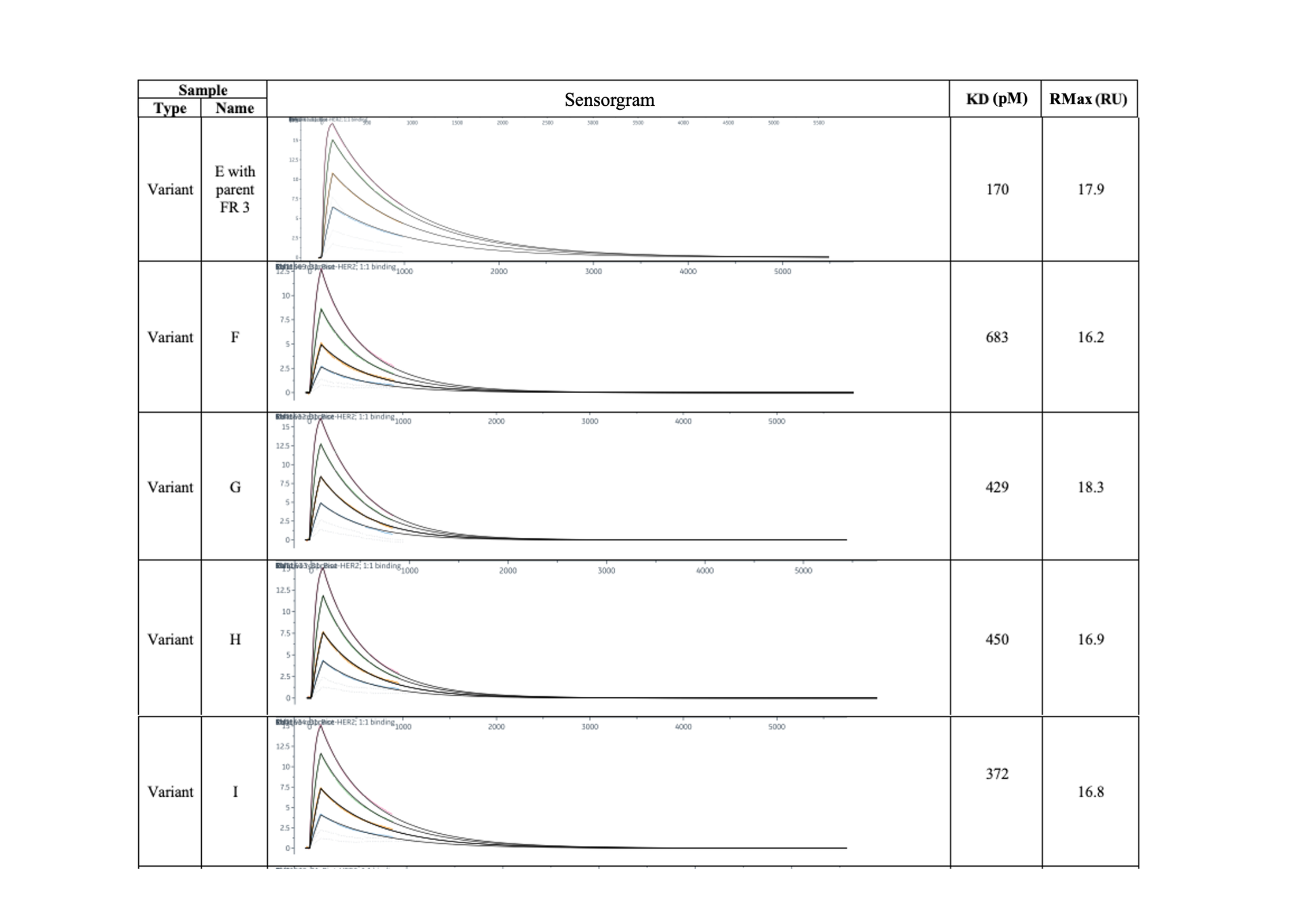

Supplement: S4 Table — (TIF) [file pone.0288259.s004.tif]

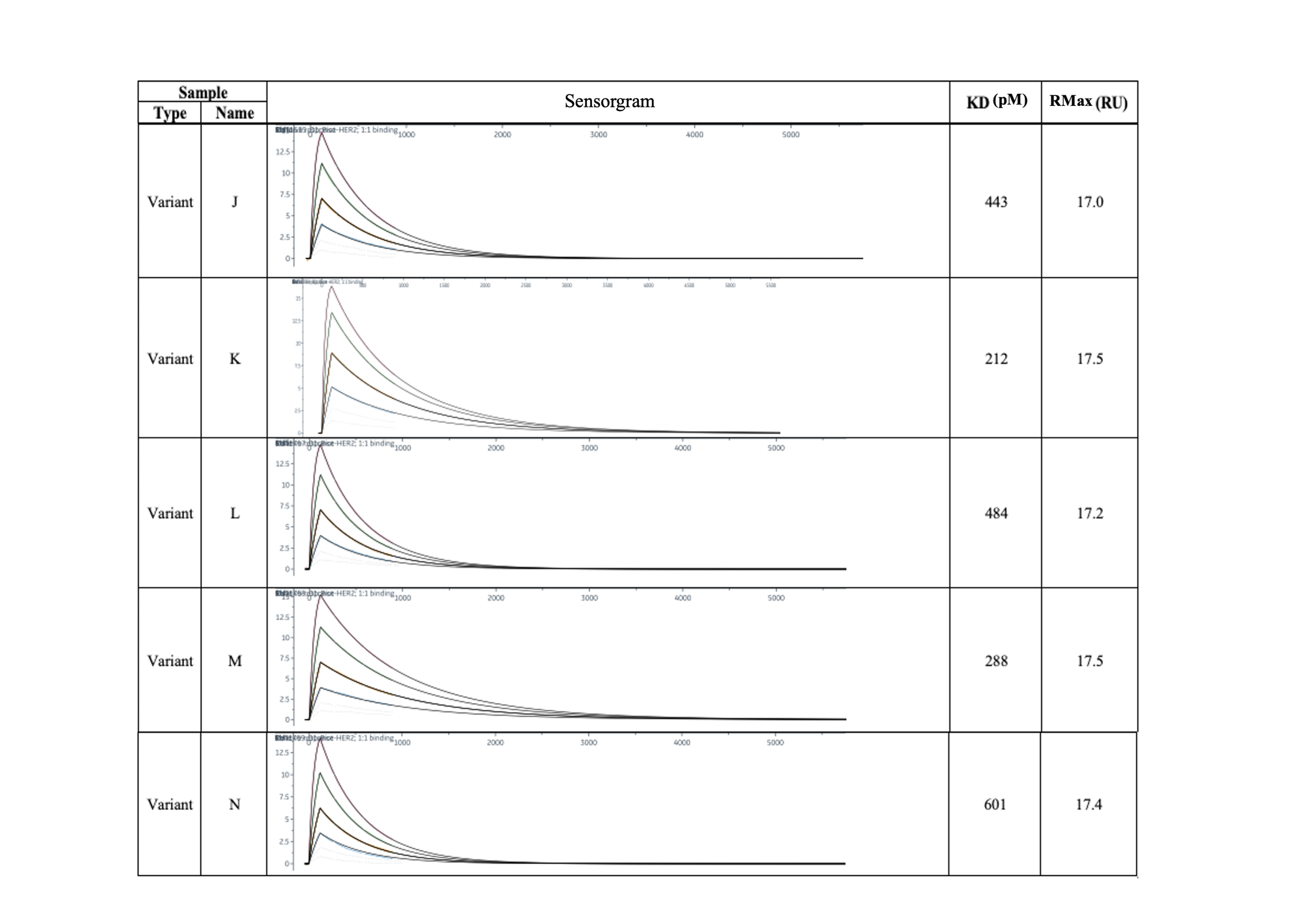

Supplement: S5 Table — (TIF) [file pone.0288259.s005.tif]
