# Supplementary material for: Co-crystallisation and humanisation of an anti-HER2 single-domain antibody as a theranostic tool
Source: PLoS One. 2023 Jul 17;18(7):e0288259. doi: 10.1371/journal.pone.0288259 (PMC10351726; doi:10.1371/journal.pone.0288259)
Supplement: Attachment — Submitted filename: Response_to_Reviewers.docx [file pone.0288259.s007.docx]

 Leicester Institute of Structural and Chemical Biology

University of Leicester

Leicester LE1 9HN

Dr Gareth Hall

Tel: +44 (0)116 229 7075

E-mail: gh126@leicester.ac.uk

20^th^ June 2023

Dr Yolanda Lemmer

Academic Editor

PLOS ONE

Dear Dr Lemmer

Please find enclosed a revised version of our paper “**Co-crystallisation and humanisation of an anti-HER2 single domain antibody as a theranostic tool”** (Manuscript Number PONE-D-23-03760). We were pleased to read that both reviewers assessed the work with great interest and that they felt the manuscript contained important information of interest to other investigators. The reviewers had highlighted several grammatical concerns that we feel we have now addressed in a revised manuscript. We have summarised the changes made to address the reviewers’ comments below and, as requested, we have provided a copy of the original manuscript with tracked changes to facilitate the review process. In addition, we have addressed the Journal requirements below, as highlighted in your last correspondence.

1. The manuscript has been adapted to comply with PLOS ONE’s style requirements, including changing font styles, reordering sections, referencing and preparing individual .TIFF files for the figures.

2. To comply with PLOS ONE’s submission requirements, in the methods section covering animal experiments we have included further details on (1) methods of sacrifice, (2) methods of anaesthesia and/or analgesia, and (3) efforts to alleviate suffering.

(1) “Mice under anaesthesia were sacrificed by cervical dislocation, after confirmation that all SPECT/CT images were technically acceptable.”

(2) “Mice were subcutaneously inoculated on the right flank with BT474 cells (6 x10^6^ cells suspended in 1:1 (v/v) matrigel:PBS) following implantation of estrogen pellets, using buprenorphine analgesic.”

“Whole-body SPECT/CT images were acquired 1.5 hr post-injection in a VECTor6-CTXUHR (MILabs) preclinical imaging system under isoflurane anaesthesia.”

(3) “The animals were observed daily for any abnormalities indicative of health problems, to prevent suffering. Only healthy animals were placed on the study.”

3. “I confirm that I have mentioned all organizations that funded my research in the Acknowledgements section of my submission, including grant numbers where appropriate.”

a) This work was funded by the research charity LifeArc, under contractual arrangement with NanoMab. LifeArc funded the research activity carried out by the University of Leicester group.

b) LifeArc and NanoMab were actively involved in the study design, data collection and analysis, as well the decision to publish and preparation of the manuscript.

c) Kovilen Sawmynaden, Sarah Davies, Richard Brown, David Tang, Maud Henry, David Tickle, David Matthews and Preeti Bakrania received a salary from LifeArc. Nicholas Wong and Hong Hoi Ting received a salary from NanoMab.

4. For the Acknowledgments section:

“I confirm that I have mentioned all organizations that funded my research in the Acknowledgements section of my submission, including grant numbers where appropriate.”

5. For the Competing Interests section:

“The authors declare the following financial interests/personal relationships which may be considered as potential competing interests: LifeArc provided the antibody humanisation service(s) under contractual arrangement with NanoMab. This does not alter our adherence to PLOS ONE policies on sharing data and materials.”

6. We have amended the authorship list to include David Tickle.

7. We have included a separate caption for each figure directly after the paragraph that first cites that figure, as highlighted in the style requirement.

8. We have included captions for the Supporting Information at the end of the manuscript, as highlighted in the style requirement.

9. We are happy that the reference list is complete and correct.

Reviewer 1

We would like to thank the reviewer for their comments. We were pleased to read that the reviewer considered the manuscript technically well-written, although they had they felt that the linguistic style contained complex and jargonistic phrases, and that these should be replaced with more generally comprehensive ones. We agree with this comment and, as such, we have adapted the manuscript accordingly. Along with the changes suggested by the reviewer, we have also rephrased several additional sentences to improve the accessibility of the manuscript. We have also reduced the number of brackets used throughout the manuscript to improve the flow and readability.

Reviewer 2

We would like to thank the reviewer for their comments. The reviewer felt that the paper was well written and well presented. We would also like to thank them for making minor suggestions regarding the use of pronouns and tenses. As a result, we have carefully ensured that the report is written in the past tense, and we have also significantly reduced the use of pronouns.

In addition to changes made in response to the reviewers’ comments, we have addressed all comments made within the ‘Revised Manuscript with Tracked Changes’ document, as well as correcting additional typographical and grammatical errors identified in the original paper. We hope that the revised paper fully addresses the concerns raised by the two reviewers and is now considered acceptable for publication in PLOS ONE.

Yours Sincerely,

Gareth Hall
