# Supplementary material for: Co-crystallisation and humanisation of an anti-HER2 single-domain antibody as a theranostic tool
Source: PLoS One. 2023 Jul 17;18(7):e0288259. doi: 10.1371/journal.pone.0288259 (PMC10351726; doi:10.1371/journal.pone.0288259)
Supplement: Attachment — Submitted filename: Manuscript-PLOSone-Reviewer Comments-Edits.docx [file pone.0288259.s006.docx]

Co-crystallisation and humanisation of an anti-HER2 single-domain antibody as a theranostic tool

Kovilen Sawmynaden^1^, Nicholas Wong^2^, Sarah Davies^1^, Richard Cowan^3^, Richard Brown^1^, David Tang^1^, Maud Henry^1^, David Matthews^1^, Mark Carr^3^, Preeti Bakrania^1^, Hong Hoi Ting^2^, Gareth Hall^3^

**Affiliations**

^1^LifeArc, Accelerator Building, Open Innovation Campus, Stevenage, SG1 2FX, United Kingdom

^2^NanoMab Rm 3-150, 3/F, China Merchants Plaza, 333 North Chengdu Road, Jingan District, Shanghai, China, 200041

^3^Department of Molecular and Cell Biology, Henry Wellcome Building, University of Leicester, University Road, Leicester, LE1 7RH, United Kingdom

**Corresponding author information to**

Gareth Hall ([gh126@leicester.ac.uk](mailto:gh126@leicester.ac.uk)); Kovilen Sawmynaden (kovilen.sawmynaden@lifearc.org)

**Keywords**

Breast cancer; erb2; Her2; antibody; sdAb; protein crystallisation

**ABSTRACT**

Human epidermal growth factor receptor-2 (HER2) is a well-recognised biomarker associated with 25 % of breast cancers. In most cases, good early detection and/or treatment correlates with an increased chance of survival. In this study, we identify and characterise a highly specific anti-HER2 single-domain antibody (sdAb), NM-02, as a potential theranostic tool. Complete structural description by X-ray crystallography reveals a non-overlapping epitope with current frontline treatment options. To reduce immunogenicity risk, we applied humanisation processes, whilst retaining wildtype-like binding properties. To further de-risk the progression towards chemistry, manufacturing and control (CMC) we performed full developability profiling revealing both thermal and physical biochemical ‘drug-like’ properties. Finally, we demonstrate the applicability of our humanised sdAb (variant k) for HER2-specific imaging purposes, using breast cancer HER2+/BT474 xenograft mice.

**INTRODUCTION**

Human epidermal growth factor receptor-2 (HER2) is a well characterised cell-surface glycoprotein (~ 185 kDa), first identified as the product of an oncogene in the late 1980s. Overexpression of HER2 has been observed in many cancers, most notably breast cancer, whereby ~ 25 % of breast cancers show a marked increase in expression levels (2 to 20-fold), correlating with higher recurrence rates and shorter relapse times (1, 2). Furthermore, overexpression of the HER2 receptor in healthy cells is able to initiate ‘cancer-like’ behaviour, including increased cell division and tumour formation (3, 4). As a result, there is strong evidence to suggest that HER2-positive (HER2+) breast cancers are more aggressive than HER2-negative (HER2-) tumours, leading to poorer patient prognosis and unfavourable tumour characteristics (1, 5, 6).

The extracellular domain (ECD) of HER2 is the target of current frontline monoclonal antibody (mAb) treatments for HER2+ breast cancer, including Trastuzumab (Herceptin®) and Pertuzumab (Perjeta®). In 2019, an antibody-drug conjugate, Trastuzumab Emtansine (Kadcyla®) was approved for adjuvant treatment of early-stage breast cancer (7). Hence, the continual development of antibody-based modalities has provided new opportunity to both treat and diagnose HER2+ tumours.

Single-domain antibodies (sdAbs or VHHs) can be isolated from the ancestral, heavy-chain-only antibodies of camelids (8). Such modalities have been shown to exhibit robust biophysical properties and can often retain antigen-specific binding activity following chemical or thermal stress (9). Additionally, their small size (~ 15 kDa) and often extended third complimentary determining region (CDR), has made them particularly attractive as diagnostic ‘tools’. This is at least in part, due to their potential to sample surfaces traditionally inaccessible to larger monoclonal antibodies (10, 11). Combining diagnostic tools with therapeutic payloads, further opens the possibility of a ‘two-in-one’ approach. This is particularly pertinent to the field of ‘Theranostics’, whereby a single modality is utilised to both stratify patient groups and subsequently deliver treatment (12). In the case of sdAbs, these can be simply appended with chemically-defined linkers and ‘loaded’ with nuclides that are either appropriate for diagnostic-based imaging (*e.g.* Technetium-99m), or cancer cell killing/therapy (*e.g.* actinium-225 or iodine 131) (11). Such a strategy allows the swapping ‘in or out’ of radiolabels, according to patient need and therefore resulting in a true personalised medicine experience.

Although sdAbs fall within the range for renal excretion (< 69 kDa) (13), repeated administration of camelid-derived biologics with low similarity to human germline (< 75 %), has the potential for increased ‘immunogenicity’ risk. Overcoming this problem is well recognised for therapeutic monoclonal antibodies (mAbs), especially those of animal origin and is generally mitigated by antibody engineering processes (14, 15). Hence, it is sensible to adopt analogous approaches for sdAbs to also minimise risk, where they might be repeatedly administered with therapeutic or theranostic purpose. In this study, we identify a highly specific anti-HER2 sdAb candidate, with a non-overlapping epitope to current frontline treatment options. Following complete epitope-paratope description, obtained by co-crystallisation with HER2-ECD, we subject the lead candidate to humanisation processes and full developability profiling in order to further de-risk progression towards chemistry, manufacturing, and control (CMC). Finally, we demonstrate the applicability of the lead humanised sdAb for HER2-specific imaging purposes, using breast cancer HER2+/BT474 xenograft mice.

**RESULTS**

***Discovery and characterisation of anti-HER2 candidate: NM-02***

A total of 40 anti-HER2 sdAbs were identified from panning an assembled phage display library (library diversity = 2 x 10^9^ colony-forming units) and comprised 19 families, based upon the amino acid sequences of CDR 1, 2 and 3. A representative subset were then triaged for affinity to target, adherence to cancer cell lines, competition with available therapeutics and off-target specificity resulting in the identification of NanoMab-02 (NM-02) as the lead candidate.

Using an enzyme-linked immunosorbent assay (ELISA) candidate NM-02 demonstrated both high affinity (EC_50_ = 1.2 nM) and specificity towards human HER2-ECD, with no detectable cross-reactivity to murine HER2-ECD (Figure 1a). The ability of NM-02 to bind human HER2 on ovarian and breast cancer cell lines was further evaluated using flow cytometry. A clear shift in fluorescence intensity was observed for HER2+ cells (BT474 and SKOV3) versus the negative control cell line (MDA-MB-231), confirming that NM-02 was able to recognise cell-surface expressed HER2 from different cancer types (Figure 1b). Competition experiments with existing HER2 therapies (Trastuzumab and Pertuzumab) were performed using surface plasmon resonance (SPR). A desirable, non-overlapping epitope for NM-02 (Figure 1c) was identified, implying that the sdAb could be utilised alongside existing treatment regimes, without steric interference.

***Off-target microarray screening***

To mitigate any future risk associated with off-target toxicity, NM-02 was subject to an extensive *in vitro* microarray accessed under commercial agreement with Retrogenix™(Freeth and Soden, 2020). Here, a dual tagged version of NM-02 (comprising a hexahistidine and hemagglutinin (HA) epitope tag was used to probe a library comprising 5484 full-length human plasma membrane proteins and cell surface-tethered, human secreted proteins. After filtering non-reproducible and/or non-specific hits, a single hit to HER2(ERBB2)/isoform 1 remained (Figure 2). Therefore, it was determined that NM-02 exhibited excellent specificity for HER2-ECD and confirmed its tractability as a drug discovery lead.

***Structure of NM-02 in complex with HER2-ECD***

To facilitate single-domain antibody engineering and epitope-paratope relationship, the structure of NM-02 in complex with HER2-ECD was solved by X-ray crystallography (PDB: 7QVK). As the epitope was initially undefined, the largest crystallisable construct of HER2-ECD, comprising residues 23-646 (UniProt ID: P04626), was pursued. Crystals of the purified complex were obtained and diffracted to 3.1 Å (Table S1). Analysis of the diffraction data indicated that the asymmetric unit contained one molecule of NM-02 in complex with one molecule HER2-ECD.

Interpretable electron density was obtained for HER2-ECD residues Gln24 to Val541 and NM-02 residues Gln3 to Ser123 (numbering residues sequentially from N- to C-terminus). Residues Leu123 to Ala132 and Ser335 to Lys336 from HER2-ECD were considered disordered and therefore not modelled. NM-02 forms a classical (variable-type) Ig-like fold, comprising of nine β-stands (Figure 3). The sequences of the three complementarity determining regions (CDR) according to the IMGT numbering system (16) are: CDR1 (Gly27-Asp38), CDR2 (Ile56-Thr65) and CDR3 (Ala105-Trp118). Two disulphide bonds (Cys23–Cys104 and Cys50–Cys112) are present. The second disulphide bond (Cys50–Cys112) covalently links framework 2 to CDR3, which itself exhibits an α-helical structure between residues Thr112.1–Trp118 (IMGT numbering) (16).

HER2-ECD comprises a four-domain architecture, as previously reported (17). Domains I-III are very similar to all published HER2 structures (max. Cα RMSD = 1.299 Å). Domain IV (residues 541–646) generally gave poor electron density and is therefore assumed to be disordered. Inspection of protein quaternary structure, reveals that NM-02 is bound to domains I and II of HER2-ECD. Assuming a classic topology, whereby domain IV is membrane proximal and precedes the transmembrane domain (18), this positions NM-02 within a potentially more accessible, membrane distal epitope (Figure 3). Notably, this is distinct from the binding sites for the anti-HER2 therapeutic antibodies Pertuzumab (19) and Trastuzumab (20), which corroborates both the applied selection criteria and obtained biochemical data (Figure 1). In addition, our HER2 epitope is different to that previously reported for an anti-HER2 camelid-derived sdAb (17).

***Features of the structural interface between NM-02 and HER2-ECD***

A total of 16 residues from NM-02 comprise the paratope (defined as < 5 Å from HER2-ECD), with 12 amino acids relinquishing at least 10 Å^2^ of solvent accessibility. It is predominantly defined by residues from CDR3, with minor contributions from CDR1 and framework1 (Figure 3). The paratope comprises a mixture of both polar and hydrophobic contacts, including the participation of four aromatic (Phe28, Tyr111, Tyr117 and Trp118) and three aliphatic side chains (Leu4, Leu111.2 and Leu115).

A total of 21 residues from domains I and II of HER2-ECD, comprise the epitope (defined as < 5 Å from NM-02) and is composed entirely of loop regions (Figure 3). Notably, it includes several basic residues (Lys170, Lys200, Arg203 and Arg217), which gives the binding site on HER2-ECD a partial positive charge. The complimentary surface of NM-02 includes a number of acidic residues (including Glu111A and Asp115), with the carboxylate sidechain of Glu111A forming a salt bridge with the guanidino moiety of Arg217 from HER2-ECD. In total there are three hydrogen bonds and one salt bridge between HER2-ECD and NM-02 that contributes to both the specificity and stability of the protein complex formed.

Overall, NM-02 buries 637 Å^2^ of surface area from bulk solvent upon complex formation and this is consistent with a mean surface area of 768.5 ± 201.0 Å^2^, as determined for this class of antibody (21). HER2-ECD buries a comparable surface area of 627 Å^2^.

***Single-domain antibody engineering: humanisation of NM-02***

Antibody humanisation is a well-established approach for de-risking any potential immunogenicity issues, that can arise as a result of utilising animal-derived variable (V) domains as the basis for human therapeutics (for review of techniques *see* Strohl and Strohl (15)). However, far less information is available with respect to strategies for humanising sdAbs. We therefore adopted a surface-editing approach, based upon methodology proposed by Vincke et al. (22) to design fourteen humanised variants (A to N) derived from the parent NM-02 (Table S2). These ranged from 77.3 % to 84.5 % human identity, as defined by comparison to nearest human germline V-gene segment (23). The primary objective was to increase the number of human-equivalent residues, whilst still retaining wild-type-like affinity, defined by us as being within 1.5-fold of that exhibited by NM-02.

Although the incorporation of human-equivalent residues into the pseudo-VH/VL interface (positions 49 and 50) of VHHs has previously been shown to be tolerable (22), we opted not to do this, as in our candidate-specific case, this would have introduced either a therapeutically undesirable deamidation motif and/or disrupted the second archetypal disulphide bond, respectively (Table S2). Instead, we inserted additional human-equivalent residues into framework 1 and 3 in order to further increase human content. Our overall strategy was therefore to design an initial set of variants (A – F) reaching desired maximal humanised content, before additional parental back mutations were incorporated (G – N), with the sole purpose of retaining wild-type-like affinity for HER2-ECD (Table S2).

***Kinetics of humanised NM-02 variants binding to HER2-ECD***

The kinetics of the interaction between NM-02 humanised variants and the extracellular domain (ECD) of HER2 was examined using SPR (Figure 4). Obtained dissociation constant (KD) for NM-02 and wild-type-like ‘framework-swapped’ controls were similar as expected and determined to be ~ 200 pM (Table S2). In contrast to that typically observed for a monoclonal antibody, NM-02 exhibits a general ‘fast-on’ and ‘fast-off’ kinetic profile, which is maintained by all humanised variants. Humanised variant K exhibits a near-equivalent dissociation constant (KD = 212 pM) to that of NM-02, while crucially maintaining an elevated human content (82.5 %). Therefore, variant K was designated as the humanised lead candidate for follow-up developability profiling.

***Developability and profiling of humanised NM-02***

Biophysical characterisation of early-stage therapeutics or diagnostic candidates is important to identify any associated risks or safety concerns, prior to beginning clinical trials (24). Therefore, we subjected the humanised lead candidate (variant K) to thermal, physical and enzymatic stress-tests, as well as solubility assessment.

The humanised lead candidate exhibited improved thermal stability (Tm = 71.6 °C) versus the NM-02 parent (Tm = 67.8 °C) (Figure 5a). Interestingly, neither molecule exhibited detectable aggregation post-complete denaturation, as confirmed by simultaneous dynamic light scattering (DLS) measurements. This may be attributed to the additional disulphide bond, which in some cases has been recognised to improve stability and reduce aggregation propensity (25).

Freeze-thaw processes are common in drug manufacturing, as sample freezing provides a means to store product, prevent microbial growth and adjust in real-time to supply and demand (26). To identify any potential liabilities associated with such stress, the humanised lead candidate was subjected to ten cycles of ‘freeze-thawing’ SEC and subsequently analysed by analytical SEC coupled to a multi-angle light scattering detector (MALS). This permitted for additional sensitivity of large aggregates, below that typically detected using UV alone. No significant increase in aggregation content was observed versus the unstressed sample (Figure 5b). The homogeneity of the sample across the main peak remained constant (polydispersity < 0.05) suggesting that despite using a simple phosphate buffered saline (PBS) formulation, the humanised lead sdAb candidate exhibited a good developability profile with respect to freeze-thaw processes.

The stability of the humanised lead candidate in sera of clinical relevance, was assessed by incubating samples in mouse, cynomolgus monkey and human serum. After 7 days (37 °C), activity to recombinant HER2-ECD was maintained in all sera (Figure 5c) with only a minimal drop-off (1.5-fold) detected in human serum. This implies generally good resistance to serum proteases, which may function to degrade and reduce efficacy within a clinical setting.

To profile the propensity for non-specific protein-protein interactions, the humanised lead candidate was subject to analytical cross-interaction chromatography (CIC). Here, samples were passed across a column pre-loaded with polyclonal immunoglobulin (Ig) and retention time (s) determined (versus an unloaded column) to derive a score (*k’*). Scores below 0.2 typically correlate to a solubility > 100 mg/mL for mAbs (27). The humanised lead candidate exhibited pass criteria similar to that of parent NM-02 and in line with that observed for a control therapeutic mAb (Figure 5d).

***SPECT/CT imaging of humanised NM-02 in HER2+/BT474 xenograft mice***

To assess the suitability of the humanised candidate (Variant K) for imaging purposes and therefore potential theranostic application, an *in vivo* biodistribution analysis was performed using orthotopic HER2+ breast cancer BT474 xenograft mice.

Single-domain antibodies were labelled with technetium-99m (^99m^Tc) and injected intravenously (IV) to assess both image quality and tumour-target specificity. Using single-photon emission computed tomography/ computed tomography (SPECT/CT) in combination with an X-ray scanner, good quality tomographic images were obtained (> 5 % ID/g) (Figure 6). Furthermore, tumour specificity was confirmed by cross-competing or ‘blocking’ of the radiolabelled response, using unlabelled sdAb, co-injected alongside. The overall performance of the humanised candidate was comparable to that of the parent, NM-02 and therefore retained all desirable *in vivo* properties, despite having undergone significant protein engineering.

**DISCUSSION**

There are over 2 million diagnoses of breast cancer per year, with a 70 - 80 % survival rate if detected early and prior to organ metastases (28, 29). Although there are different and complex underlying mechanisms, HER2-positive breast cancer prevalence is estimated to be between 15 and 19 % (30). In most cases, good early detection correlates with an increased chance of survival.

Here we describe the discovery, biochemical characterisation, structural determination, humanisation and *in vivo* efficacy of NM-02, in order to demonstrate its tractability as a potential theranostic tool. The sdAb is highly-specific for HER2-ECD, recognising a distinct membrane-distal epitope, which is non-overlapping with current front-line therapeutics. Hence, combinatorial approaches including NM-02 could be further explored, especially where Trastuzumab resistance is a recognised issue (31).

Regulatory precedence for humanisation of sdAbs is set by Calplacizumab (Cablivi), the only FDA-approved sdAb therapy available (Elverdi and Eskazan, 2019). Unlike some other humanised sdAbs, we have not used a pre-humanised scaffold as an acceptor for CDR-grafting (32). Instead, we embarked on a bespoke humanisation approach which allowed us to maximise the retention of wild-type properties, whilst achieving increased human content. For example, sub-nanomolar potency and good SPECT/CT image quality of the humanised lead candidate (variant K) has been retained. Lastly, extensive stress-testing and developability profiling reveals desirable antibody drug-like properties, which further de-risks our candidate for progression along the clinical stage pipeline and its use as a potential anti-HER2 theranostic tool.

**MATERIALS AND METHODS**

***Immunisation and phage library construction***

Recombinant HER2-ECD (produced in HEK cells and ≥ 90 % via SDS-PAGE) was used to immunise two domestic camels (*Camelus bactrianus*) over a period of several weeks. Peripheral blood lymphocytes (PBL) were harvested, ribonucleic acid (RNA) extracted and VHH genes amplified to construct a phage display library. Incorporation of relevant sized inserts was confirmed using polymerase cain reaction) (PCR) and diversity determined by measuring colony-forming units (CFU).

***Cross-reactivity testing of NM-02 to recombinant HER2-ECDs***

ELISA microplates were coated with human HER2-ECD-Fc or mouse HER2-ECD-Fc (R&D systems, Minneapolis, MN) and blocked with 1 % bovine serum albumin (BSA) at room temperature. Single-domain antibody NM-02 was serially diluted (from 160 nM) and added to the microplate, before binding was probed using a mouse anti-HA secondary antibody (Biolegend, San Diego, CA, USA). An anti-mouse IgG-alkaline phosphatase (Proteintech, Rosemont, IL, USA) in the presence of TMB (eBioscience, San Diego, CA, USA) was used for colorimetric detection. Absorbance at 450 nm was recorded on a microplate reader (Bio-Tek).

***Cell-surface binding of NM-02 to cancer cell lines***

Adherent cancer cell lines (BT474, SKOV3 and MDA-MB-231) were detached from flasks using a non-enzymatic cell dissociation buffer (Cell Stripper®; Corning, NY, USA) and re-suspended in PBS (2x10^6^ cells/mL). Cells were first incubated with FcR blocking reagent (Miltenyi Biotec, Germany) followed by NM-02 and Alexa Fluor 488 anti-HA antibody (Biolegend, San Diego, CA, USA). All incubations were performed at 4 ºC in the dark and cells were washed twice with PBS between each step. Flow cytometry analyses were performed on a FACSCalibur™ flow cytometer (BD Biosciences, UK).

***Competition of NM-02 with Trastuzumab and Pertuzumab***

Human HER2 ECD-Fc was coupled to a CM5 sensor chip (800 resonance units) pre-loaded into Biacore® 3000 system (Cytiva). NM-02, Trastuzumab (Roche) or Pertuzumab (Roche) were sequentially injected either alone or in combination at 30 µL/min in HBS-EP buffer in two 180s binding phases. The curves were analysed using BIAevaluation (version 4.1) and visually interpreted.

***Microarray screening***

Microarray library screening was performed by Retrogenix ([www.retrogenix.com](http://www.retrogenix.com)) and comprised fixed HEK293 cells overexpressing 5484 full-length plasma membrane and membrane-tethered secretory proteins. Recombinant NM-02 (2 µg/mL) was either HA- and/or His-tagged and ‘hits’ detected using anti-tag secondary antibodies conjugated to AlexaFluor 647. For additional assay details *see* Freeth and Soden (33).

***Production of humanised single-domain antibodies***

DNA inserts were codon-optimised (*Cricetulus griseus*), synthesised and subcloned into pcDNA3.1 (+) by GenScript. A human Ig-*k* (IGKV3D-15) leader sequence was incorporated upstream of the coding sequences, for secretory expression. A His-tag (including a short GSG linker) was incorporated at the C-terminus to facilitate protein purification.

Prepared DNA plasmids were transiently transfected into ExpiCHO-S™ cells and cultured according to the manufacturer’s instructions (Thermo Fisher Scientific). Supernatants were harvested by centrifugation, filter-sterilised (Stericup®; Merck) and supplemented with 25 mM HEPES (pH 7.2) and 0.05 % sodium azide. Harvested supernatants were stored at 4 °C. Single-domain antibodies were purified from the supernatant by affinity chromatography (HisTrap™ Excel column; Cytiva) and size-exclusion (HiLoad (16/600) Superdex 75; Cytiva) chromatography, into a final buffer of PBS (10 mM sodium phosphate, pH 7.5, and 150 mM NaCl).

All samples were quality-controlled for aggregation (< 5 %) using an analytical SEC column (AdvanceBio SEC 130Å) fitted to a 1260 Infinity HPLC system (Agilent) and coupled in series to a multi-angle static light scattering (MALS) detector and differential refractometer (Wyatt).

Endotoxin measurements were performed using a Endosafe® nexgen-PTS™ device (Charles River).

***Production of recombinant HER2-ECD for crystallisation***

Expression and purification of HER2-ECD (23-646; UniProt ID P04626) was performed by Proteros ([www.proteros.com](http://www.proteros.com)). The protein was expressed in HEK293F cells and purified by a multi-step chromatographic procedure, including affinity capture *via* Ni-NTA, deglycosylation and size-exclusion chromatography. Lastly, the sample was concentrated to 18 mg/mL, flash-frozen in liquid nitrogen and stored at minus 80 ^o^C. The storage buffer comprised: 20 mM HEPES, pH 7.0, and 150 mM NaCl.

***Crystallisation of NM-02 bound to HER2-ECD***

A protein complex, comprising NM-02 and HER2-ECD was assembled by mixing the -sdAb and target ligand at a molar ratio of 2:1. The mixture was incubated for 45 minutes at 4 °C, concentrated using a centrifugal concentrator (Vivaspin-20; Sartorius) to a final volume of 2 mL and purified by size-exclusion chromatography (Superdex 200 10/300 column; Cytiva). The mobile phase comprised 20 mM Tris-HCl, pH 7.5, and 50 mM NaCl. Fractions corresponding to the protein complex peak were pooled and re-concentrated (Vivaspin-20; Sartorius) to 4 mg/mL.

Initial crystallisation trials were performed using sitting drop vapor diffusion and 96-well block screens (Molecular Dimensions) at 20 ^o^C. Optimal crystals grew in 13 % PEG6000, 0.1 M MES (pH 6.5) and 7 % 2-methyl-2,4-pentanediol. A cryoprotectant consisting of 20 % 2-methyl-2,4-pentanediol (v/v) in mother liquor was used. X-ray diffraction data were collected at the Diamond Light Source (Oxford, UK) using beamline I24.

Diffraction data were indexed and integrated in xds (34) and scaled to 3.1 Å using AIMLESS as part of CCP4 (35). A Matthews Coefficient (36) calculated one molecule of NM-02 and one molecule of HER2-ECD present in the asymmetric unit, giving a solvent content of 67 %. A homology model of NM-02 was generated with SWISS-MODEL (37). The structure of the NM-02 in complex with HER2-ECD was determined by molecular replacement, using the program PHASER (38) input with the structures of HER2-ECD (PDB ID 5MY6) and the NM-02 homology model used in the search. Model building and structural refinement was carried out with Coot (39) and REFMAC 5.0 (40) using restrained refinement and isotropic B-factors. X-ray data collection and refinement statistics are given in Table S1.

***Binding kinetics of humanised single-domain antibody variants to HER2-ECD***

HER2-ECD biotinylated at single site (Sino Biological) was applied to a streptavidin (SA) sensor chip (0.1 µg/mL) pre-loaded into a Biacore 8 K instrument (Cytiva). The flow rate and temperature were set to 100 µL/min and 25 ℃, respectively. All experiments were performed in HBS-EP+ buffer. Single-domain antibodies were injected in a multi-cycle kinetics method using a 2-fold dilution series starting at a top concentration of 5 nM, including replicates (Rmax = 15-20 RU). Data was processed using Biacore Insight software and fitted to a 1:1 model.

***Melting temperature (Tm) measurements***

Single-domain antibody samples (1 mg/mL) were loaded (in triplicate) into an Uncle instrument (Unchained Labs). Samples were thermally ramped (0.5 ℃/min) and transiently held (120 sec) at discrete temperature points between 25 ℃ and 95 ℃. A complete fluorescence spectrum (250-720 nm) was captured for each sample at every temperature point and changes monitored using the Barycentric mean (BCM). Melting temperatures were determined by identifying the midpoint of the fluorescence transition (dy/dx = 0). Static light scattering (SLS) was measured at 266 nm and 473 nm.

***Freeze-thaw stress-testing***

Single-domain antibody samples were concentrated to 7.5 mg/mL and subject to ten freeze-thaw cycles by placing at minus 80 ℃ (15 mins) followed by room temperature thawing (15 mins). Aggregation content was determined by analytical SEC-MALS, as described above.

***Serum stability***

Single-domain antibody samples (His-tagged) were added in duplicate to a 96-well plate, containing either human, mouse or cynomolgus sera (supplied by BioIVT) and incubated for 7 days at 37 ℃ (final concentration = 100 µg/mL). Retention of binding activity was determined by ELISA versus single-domain antibody samples incubated in PBS, under the same conditions. The ELISA set-up comprised recombinant human HER2-Fc (1 µg/mL) coated to the surface of a MaxiSorp™ 384 well plate (Nunc). Sera were serially diluted, applied and binding probed using an anti-HIS-HRP secondary antibody conjugate (in the presence of TMB substrate). Absorbance was measured at 650 nm and data fit using non-linear regression to obtain half-maximal effective concentrations (EC_50_)

***Cross-interaction chromatography (CIC)***

A (1 mL) HiTrap NHS-activated column (Cytiva) was loaded with ≥ 10 mg human polyclonal IgG as a coupling ligand. A control column was prepared in the same way, but without ligand loading. Single-domain antibody samples (0.5 mg/mL) were injected separately onto each column and a retention factor (*k’*) determined according to:

*k’* = [(*Tr* – *Tm*)]/*Tm*

where *Tr* is the retention time of the sample on the polyclonal IgG column and *Tm* is the retention time on the control column. Data was acquired on a 1260 Infinity HPLC system (Agilent) using a mobile phase comprising PBS (+ 0.01 % NaN_3_) and a constant flow-rate (0.1 mL/min.).

***SPECT/CT imaging and HER2 positive BT474 xenograft mouse model***

Six-week-old, female Balb/C nu/nu mice (Charles River) were subcutaneously inoculated on the right flank with BT474 cells (6 x10^6^ cells suspended in 1:1 (v/v) matrigel:PBS) following implantation of estrogen pellets. Tumour growth and animal body weight were monitored twice a week. Tumour size was calculated according to:

Tumour size = 0.52*length*width^2^

Approximately three to four weeks after inoculation, when the average xenograft size had reached 200-300 mm^3^, mice were randomly assigned to four groups for *in vivo* imaging. Mice in group A (n=5) were intravenously administered 15.3 ± 2.6 MBq of [^99m^Tc]-NM-02 (equivalent to 20 µg of radiolabelled NM-02). Mice in group B (n=5) were also intravenously administered 16.3 ± 2.6 MBq of [^99m^Tc]-NM-02, plus an additional 180 µg of unlabelled (or ‘cold’) NM-02. Mice in group C (n=5) were intravenously administered 19.4 ± 0.5 MBq of [^99m^Tc]-Variant K (equivalent to 20 µg of radiolabelled humanised variant K). Mice in group D (n=5) were also intravenously administered 23.5 ± 1.0 MBq of [^99m^Tc]-Variant K, plus an additional 180 µg of unlabelled (or ‘cold’) humanised variant K. Whole-body SPECT/CT images were acquired 1.5 hr post-injection in a VECTor6-CTXUHR (MILabs) preclinical imaging system under isoflurane anaesthesia. Biodistribution of [^99m^Tc]-NM-02 and Variant K was calculated based on SPECT/CT images. Biodistribution at 1.5 hr post injection for different groups was compared by two-sample unequal variance t-tests. All animal studies were performed under approval by Queen Mary University of London Project Assessment Committee under the UK Home Office.

**Data availability**

The crystal structure of NM-02 in complex with HER2-ECD has been deposited in the protein data bank (PDB ID: 7QVK). All other data is deposited within the manuscript.

**Supporting information**

This article contains supporting information.

**Acknowledgments**

The authors would like to acknowledge the efforts of our service contract partners at Retrogenix and Invicro.

**Funding**

LifeArc provided humanisation service(s) to NanoMab under contractual arrangement. LifeArc is a self-funded medical charity (registered with the Charity Commission for England and Wales no. 1015243 and a charity registered in Scotland with the Office of the Scottish Charity Regulator no. SC037861).

**REFERENCES**

1.	Hicks DG, Kulkarni S. HER2+ breast cancer: review of biologic relevance and optimal use of diagnostic tools. Am J Clin Pathol. 2008;129(2):263-73.

2.	Slamon DJ, Clark GM, Wong SG, Levin WJ, Ullrich A, McGuire WL. Human breast cancer: correlation of relapse and survival with amplification of the HER-2/neu oncogene. Science. 1987;235(4785):177-82.

3.	Di Fiore PP, Pierce JH, Kraus MH, Segatto O, King CR, Aaronson SA. erbB-2 is a potent oncogene when overexpressed in NIH/3T3 cells. Science. 1987;237(4811):178-82.

4.	Pegram MD, Konecny G, Slamon DJ. The molecular and cellular biology of HER2/neu gene amplification/overexpression and the clinical development of herceptin (trastuzumab) therapy for breast cancer. Cancer Treat Res. 2000;103:57-75.

5.	Ariga R, Zarif A, Korasick J, Reddy V, Siziopikou K, Gattuso P. Correlation of her-2/neu gene amplification with other prognostic and predictive factors in female breast carcinoma. Breast J. 2005;11(4):278-80.

6.	Traina A, Agostara B, Marasa L, Calabro M, Zarcone M, Carruba G. HER2/neu expression in relation to clinicopathologic features of breast cancer patients. Ann N Y Acad Sci. 2006;1089:159-67.

7.	Girish S, Gupta M, Wang B, Lu D, Krop IE, Vogel CL, et al. Clinical pharmacology of trastuzumab emtansine (T-DM1): an antibody-drug conjugate in development for the treatment of HER2-positive cancer. Cancer Chemother Pharmacol. 2012;69(5):1229-40.

8.	Hamers-Casterman C, Atarhouch T, Muyldermans S, Robinson G, Hamers C, Songa EB, et al. Naturally occurring antibodies devoid of light chains. Nature. 1993;363(6428):446-8.

9.	Dumoulin M, Conrath K, Van Meirhaeghe A, Meersman F, Heremans K, Frenken LGJ, et al. Single-domain antibody fragments with high conformational stability. Protein Sci. 2002;11(3):500-15.

10.	Desmyter A, Transue TR, Ghahroudi MA, Thi MH, Poortmans F, Hamers R, et al. Crystal structure of a camel single-domain VH antibody fragment in complex with lysozyme. Nat Struct Biol. 1996;3(9):803-11.

11.	Yang EY, Shah K. Nanobodies: Next Generation of Cancer Diagnostics and Therapeutics. Frontiers in Oncology. 2020;10(1182).

12.	Hapuarachchige S, Artemov D. Theranostic Pretargeting Drug Delivery and Imaging Platforms in Cancer Precision Medicine. Frontiers in Oncology. 2020;10(1131).

13.	Zhao L, Ren T-h, Wang DD. Clinical pharmacology considerations in biologics development. Acta Pharmacologica Sinica. 2012;33(11):1339-47.

14.	Harding FA, Stickler MM, Razo J, DuBridge R. The immunogenicity of humanized and fully human antibodies. MAbs. 2010;2(3):256-65.

15.	Strohl WR, Strohl LM. Variable chain engineering – humanization and optimization approaches. Therapeutic Antibody Engineering: Woodhead Publishing; 2012. p. 111-595.

16.	Lefranc M-P, Pommié C, Ruiz M, Giudicelli V, Foulquier E, Truong L, et al. IMGT unique numbering for immunoglobulin and T cell receptor variable domains and Ig superfamily V-like domains. Developmental & Comparative Immunology. 2003;27(1):55-77.

17.	D'Huyvetter M, De Vos J, Xavier C, Pruszynski M, Sterckx YGJ, Massa S, et al. (131)I-labeled Anti-HER2 Camelid sdAb as a Theranostic Tool in Cancer Treatment. Clin Cancer Res. 2017;23(21):6616-28.

18.	Ou S-HI, Schrock AB, Bocharov EV, Klempner SJ, Haddad CK, Steinecker G, et al. HER2 Transmembrane Domain (TMD) Mutations (V659/G660) That Stabilize Homo- and Heterodimerization Are Rare Oncogenic Drivers in Lung Adenocarcinoma That Respond to Afatinib. Journal of Thoracic Oncology. 2017;12(3):446-57.

19.	Franklin MC, Carey KD, Vajdos FF, Leahy DJ, de Vos AM, Sliwkowski MX. Insights into ErbB signaling from the structure of the ErbB2-pertuzumab complex. Cancer Cell. 2004;5(4):317-28.

20.	Cho HS, Mason K, Ramyar KX, Stanley AM, Gabelli SB, Denney DW, Jr., et al. Structure of the extracellular region of HER2 alone and in complex with the Herceptin Fab. Nature. 2003;421(6924):756-60.

21.	Mitchell LS, Colwell LJ. Analysis of nanobody paratopes reveals greater diversity than classical antibodies. Protein Eng Des Sel. 2018;31(7-8):267-75.

22.	Vincke C, Loris R, Saerens D, Martinez-Rodriguez S, Muyldermans S, Conrath K. General strategy to humanize a camelid single-domain antibody and identification of a universal humanized nanobody scaffold. J Biol Chem. 2009;284(5):3273-84.

23.	Ehrenmann F, Lefranc M-P. IMGT/DomainGapAlign: IMGT Standardized Analysis of Amino Acid Sequences of Variable, Constant, and Groove Domains (IG, TR, MH, IgSF, MhSF). Cold Spring Harbor Protocols. 2011;2011(6):pdb.prot5636.

24.	Jain T, Sun T, Durand S, Hall A, Houston NR, Nett JH, et al. Biophysical properties of the clinical-stage antibody landscape. Proceedings of the National Academy of Sciences. 2017;114(5):944-9.

25.	Kunz P, Zinner K, Mücke N, Bartoschik T, Muyldermans S, Hoheisel JD. The structural basis of nanobody unfolding reversibility and thermoresistance. Scientific Reports. 2018;8(1):7934.

26.	Kolhe P, Badkar A. Protein and solute distribution in drug substance containers during frozen storage and post-thawing: A tool to understand and define freezing–thawing parameters in biotechnology process development. Biotechnology Progress. 2011;27(2):494-504.

27.	Jacobs SA, Wu S-J, Feng Y, Bethea D, O’Neil KT. Cross-Interaction Chromatography: A Rapid Method to Identify Highly Soluble Monoclonal Antibody Candidates. Pharmaceutical Research. 2009;27(1):65.

28.	Bray F, Ferlay J, Soerjomataram I, Siegel RL, Torre LA, Jemal A. Global cancer statistics 2018: GLOBOCAN estimates of incidence and mortality worldwide for 36 cancers in 185 countries. CA Cancer J Clin. 2018;68(6):394-424.

29.	Harbeck N, Penault-Llorca F, Cortes J, Gnant M, Houssami N, Poortmans P, et al. Breast cancer. Nature Reviews Disease Primers. 2019;5(1):66.

30.	Cronin KA, Harlan LC, Dodd KW, Abrams JS, Ballard-Barbash R. Population-based estimate of the prevalence of HER-2 positive breast cancer tumors for early stage patients in the US. Cancer Invest. 2010;28(9):963-8.

31.	Pohlmann PR, Mayer IA, Mernaugh R. Resistance to Trastuzumab in Breast Cancer. Clinical Cancer Research. 2009;15(24):7479-91.

32.	Yu Y, Li J, Zhu X, Tang X, Bao Y, Sun X, et al. Humanized CD7 nanobody-based immunotoxins exhibit promising anti-T-cell acute lymphoblastic leukemia potential. Int J Nanomedicine. 2017;12:1969-83.

33.	Freeth J, Soden J. New Advances in Cell Microarray Technology to Expand Applications in Target Deconvolution and Off-Target Screening. SLAS Discov. 2020;25(2):223-30.

34.	Kabsch W. Integration, scaling, space-group assignment and post-refinement. Acta Crystallographica Section D. 2010;66(2):133-44.

35.	Winn MD, Ballard CC, Cowtan KD, Dodson EJ, Emsley P, Evans PR, et al. Overview of the CCP4 suite and current developments. Acta Crystallogr D Biol Crystallogr. 2011;67(Pt 4):235-42.

36.	Kantardjieff KA, Rupp B. Matthews coefficient probabilities: Improved estimates for unit cell contents of proteins, DNA, and protein-nucleic acid complex crystals. Protein Sci. 2003;12(9):1865-71.

37.	Schwede T, Kopp Jr, Guex N, Peitsch MC. SWISS-MODEL: an automated protein homology-modeling server. Nucleic Acids Research. 2003;31(13):3381-5.

38.	McCoy AJ, Grosse-Kunstleve RW, Adams PD, Winn MD, Storoni LC, Read RJ. Phaser crystallographic software. Journal of Applied Crystallography. 2007;40(4):658-74.

39.	Emsley P, Cowtan K. Coot: model-building tools for molecular graphics. Acta Crystallogr D Biol Crystallogr. 2004;60(Pt 12 Pt 1):2126-32.

40.	Murshudov GN, Skubak P, Lebedev AA, Pannu NS, Steiner RA, Nicholls RA, et al. REFMAC5 for the refinement of macromolecular crystal structures. Acta Crystallographica Section D. 2011;67(4):355-67.
